# ATP Synthase Diseases of Mitochondrial Genetic Origin

**DOI:** 10.3389/fphys.2018.00329

**Published:** 2018-04-04

**Authors:** Alain Dautant, Thomas Meier, Alexander Hahn, Déborah Tribouillard-Tanvier, Jean-Paul di Rago, Roza Kucharczyk

**Affiliations:** ^1^Institut de Biochimie et Génétique Cellulaires, Centre National de la Recherche Scientifique UMR 5095, Université de Bordeaux, Bordeaux, France; ^2^Department of Life Sciences, Imperial College London, London, United Kingdom; ^3^Department of Structural Biology, Max-Planck-Institute of Biophysics, Frankfurt, Germany; ^4^Institute of Biochemistry and Biophysics, Polish Academy of Sciences, Warsaw, Poland

**Keywords:** mitochondrial diseases, F_1_F_o_ ATP synthase structure, mitochondrial DNA (mtDNA), MT-ATP6, MT-ATP8

## Abstract

Devastating human neuromuscular disorders have been associated to defects in the ATP synthase. This enzyme is found in the inner mitochondrial membrane and catalyzes the last step in oxidative phosphorylation, which provides aerobic eukaryotes with ATP. With the advent of structures of complete ATP synthases, and the availability of genetically approachable systems such as the yeast *Saccharomyces cerevisiae*, we can begin to understand these molecular machines and their associated defects at the molecular level. In this review, we describe what is known about the clinical syndromes induced by 58 different mutations found in the mitochondrial genes encoding membrane subunits *8* and *a* of ATP synthase, and evaluate their functional consequences with respect to recently described cryo-EM structures.

## Introduction

Mitochondria support aerobic respiration and produce the bulk of cellular ATP by oxidative phosphorylation (OXPHOS) (Saraste, 1999). Electrons provided by the oxidation of fatty acids and carbohydrates are shuttled to oxygen along four respiratory chain (RC) complexes (I–IV) embedded in the inner mitochondrial membrane (IMM), producing water and releasing the energy necessary to pump protons from the mitochondrial matrix to the intermembrane space (IMS). This results in the formation of transmembrane electrochemical ion gradient across the IMM, also called the proton-motive force (*pmf*). The outer side of the IMM is positively charged (the *p*-side) while the inner side is negatively charged (the *n*-side). The *pmf* enables the F_1_F_o_ ATP synthase to produce ATP from ADP and inorganic phosphate (Boyer, 1997). The OXPHOS complexes contain ~90 structural proteins of which 13 are encoded by the mtDNA in humans.

More than 150 distinct genetic mitochondrial dysfunction syndromes characterized by a diminished OXPHOS capacity have been described (Tuppen et al., 2010; Hejzlarova et al., 2014; Chinnery, 2015; Ng and Turnbull, 2015; Stewart and Chinnery, 2015; Xu et al., 2015). These diseases affect at least 1 in 5,000 live human births (Skladal et al., 2003). Typical clinical traits include visual/hearing defects, encephalopathies, cardiomyopathies, myopathies, diabetes, liver, and renal dysfunctions (Table 1; Dimauro and Schon, 2003; Zeviani and Carelli, 2007; Vafai and Mootha, 2012). Many known cases result from alterations in mtDNA (~15%, e.g., NARP, MILS, LHON), which occur as a result of this DNA's high susceptibility to mutations because of the nearby ROS production and the poor effectiveness of the mitochondrial DNA repair system (Wallace, 2010). More than 600 different point mutations and innumerable large-scale rearrangements of mtDNA have been implicated in human diseases (Lott et al., 2013).

**Table 1:** Diseases and syndromes caused by mutations in ATP8 and ATP6.

**Disease/Syndrome**	**Phenotypes**
Apical hypertrophic cardiomyopathy (AHCM) and neuropathy	primary disease of the myocardium (the muscle of the heart) in which a portion of the myocardium is hypertrophied (thickened) without any obvious cause, creating functional impairment of the cardiac muscle; neuropathy is damage to or disease affecting nerves, which may impair sensation, movement, gland or organ function, or other aspects of health, depending on the type of nerve affected
Ataxia	genetic disorders characterized by slowly progressive incoordination of gait and is often associated with poor coordination of hands, speech, and eye movements, with full mental capacity
Autism	neurodevelopmental disorder, characterized by impaired social interaction, verbal and non-verbal communication, and restricted and repetitive behavior; noticeably affected by mitochondrial dysfunction which impairs energy metabolism
Charcot-Marie-Tooth syndrome (CMT)	hereditary disorders that damage the nerves in arms and legs (peripheral nerves); symptoms usually begin in feet and legs, but they may eventually affect hands and arms
Encephalopathy	abnormal brain function or brain structure, symptoms may be mental or physical dysfunctions, depending on what part of the brain is being affected
Epilepsy with Brain Pseudoatrophy	brain disorder manifesting by seizures, dementia, convulsions, loss of control on muscles, difficulties with talking
Episodic Weakness	muscular disorders (myopathies) that are only present after exercise or are exacerbated by exercise, skeletal muscle diseases, may be accompanied by neurological symptoms
Hereditary Spastic Paraplegia (HSP)	heterogeneous disorder characterized by lower extremity spasticity and weakness
Familial Bilateral Striatal Necrosis (FBSN)	acute neurological syndrome associated with radiological findings, respiratory illnesses presenting with an array of neurological findings, including axial ataxia, grimacing, mutism, head nodding, and high-pitched cry
Infantile cardiomyopathy	severe, eventually fatal, cardiac arrhythmias, characterized pathologically by cardiac hypertrophy and by a distinctive type of focal degeneration of the muscle cells, which lose their myofibrils, undergo marked mitochondrial hyperplasia, become rounded in shape and enlarged, and resemble histiocytes
Leber Hereditary Optic Neuropathy (LHON)	maternally inherited disease leading to acute bilateral blindness due to loss of the optic nerve and papillomacular bundle nerve fibers, predominantly in young men
Left Ventricular HyperTrabeculation syndrome (LVHT) (noncompaction)	myocardial abnormality of the apex, characterized by multiple, myocardial cotyledo-like protrusions and interwoven strings, all lined by endocardium; in three quarters of the cases associated with neuromuscular disorders
Maternally Inherited Diabetes and Deafness syndrome (MIDD)	form of diabetes that is often accompanied by hearing loss, especially of high tones, characterized by high blood sugar levels (hyperglycemia) resulting from a shortage of the hormone insulin
Maternally Inherited Leigh Syndrome (MILS)	early-onset progressive neurodegenerative disorder with a characteristic neuropathology consisting of focal, bilateral lesions in one or more areas of the central nervous system, manifesting with (encephalopathy), lactic acidosis, seizures, heart disease (cardiomyopathy), breathing (respiratory) abnormalities, and developmental delays
Mesial Temporal Lobe Epilepsies with Hippocampal Sclerosis (MTLE-HS)	chronic neurological condition characterized by recurrent, unprovoked epileptic seizures (epilepsy) which originate in the temporal lobe of the brain, its pathophysiological substrate is usually hippocampal sclerosis, the most common epileptogenic lesion encountered in patients with epilepsy
Metabolic Syndrome (MS)	disorder characterized by a group of metabolic abnormalities including hyperglycemia, hypertension, hyperlipaemia and central obesity, which are the risk factors of cardiovascular disease and diabetes
Motor Neuron Syndrome (MNS)	cognitive impairment, exercise intolerance, and progressive muscle weakness
Myopathy, Lactic Acidosis, and Sideroblastic Anemia (MLASA)	mitochondrial disorder specific to skeletal muscle and bone marrow with myopathy, lactic acidosis, and sideroblastic anemia with ringed sideroblasts
Neurogenic Ataxia Retinis Pigmentosa syndrome (NARP)	mitochondrial disease affecting nervous system manifesting with pain in the arms and legs (sensory neuropathy), muscle weakness, problems with balance and coordination (ataxia), vision loss caused by retinitis pigmentosa or changes in the light-sensitive tissue that lines the back of the eye
Periodic paralyzes	genetic diseases that lead to weakness or paralysis (rarely death) from common triggers such as cold, heat, high carbohydrate meals, not eating, stress or excitement, and physical activity of any kind
Schizophrenia	mental disorder, often characterized by abnormal social behavior and failure to recognize what is real
SpinoCerebellar Ataxia (SCA)	ataxia that is due to dysfunction of the cerebellum
Tetralogy of Fallot (ToF)	type of congenital heart disease, essentially a right-sided heart disease, with characteristic features of ventricular septal defect, right ventricular outflow tract obstruction, aortic dextroposition, and right ventricular hypertrophy

This review focuses on mutations in the MT-ATP8 and MT-ATP6 genes (further named ATP8 and ATP6) encoding subunits *8* and *a* of ATP synthase, respectively, that were identified in patients with various disorders. We summarize what is known about their clinical and functional consequences. Based on recent high-resolution structures (Morales-Rios et al., 2015a; Zhou et al., 2015; Hahn et al., 2016; Guo et al., 2017), we define the topological locations of these mutations, which helps understand their impact on ATP synthase structure, function and assembly.

## ATP synthase structure and function

**Figure 1:**
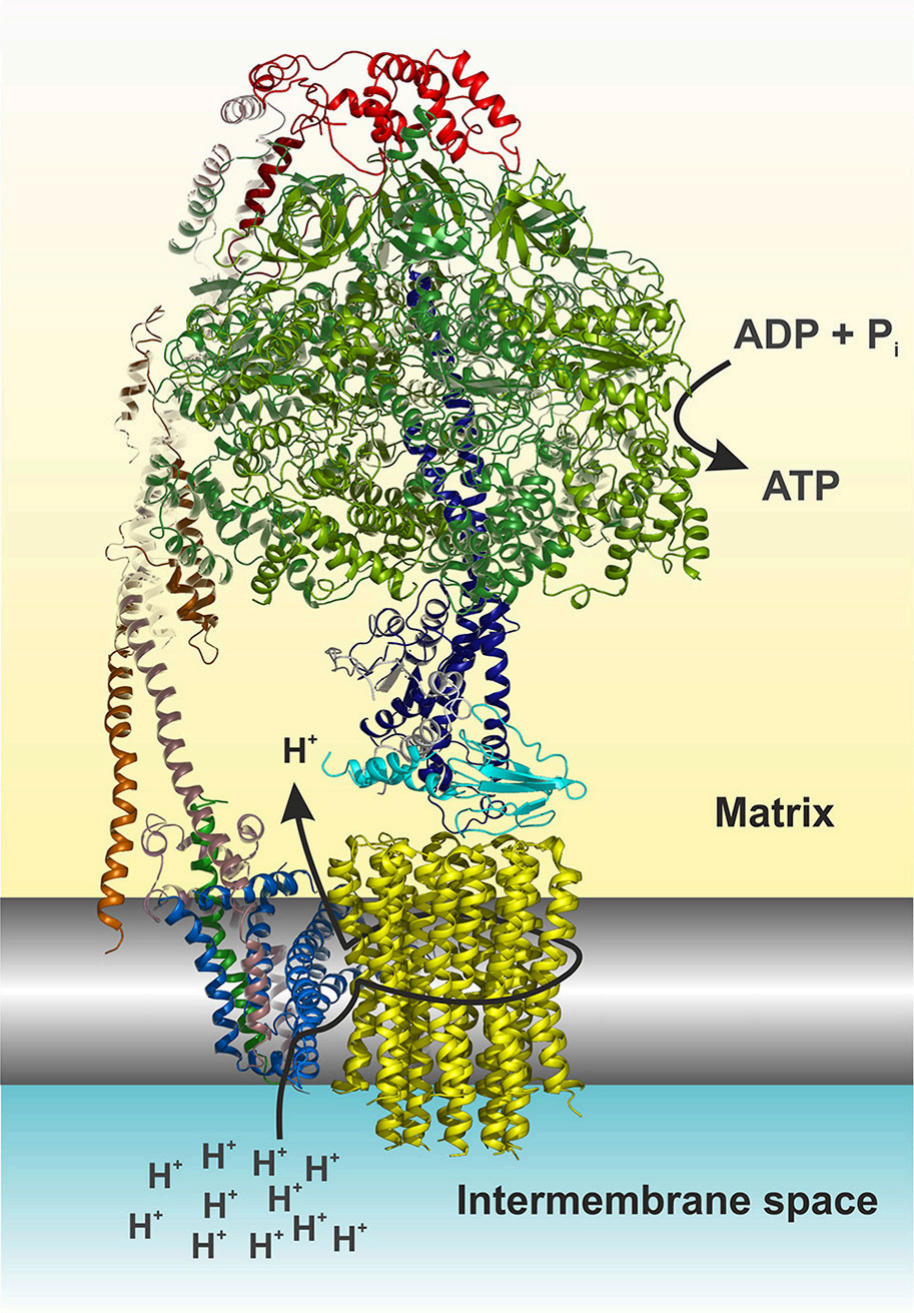
Cartoon representation of the yeast F_1_F_o_ ATP synthase. The view is horizontally to the membrane shown in grayscale. The structure was drawn according to (Hahn et al., 2016, PDB code 5FL7). For simplicity the structure is shown without subunits *e, f*, *g, I*, and a truncated subunit *b*. The figure was made in Pymol (The PyMOL Molecular Graphics System, Version 0.99, Schrödinger, LLC), using the following color code: α, forest; β, split pea; γ, density; δ, cyan; ε, white; *OSCP*, red; *b* (= *4* in yeast), dirty violet; *d*, orange; *h*, salmon; *8*, green; *a*, blue; *c*-ring, yellow. The arrows indicate the path of protons (see also Figure 4) and nucleotide conversion. For details see text.

Mitochondrial ATP synthase is a unique macromolecular rotary machine of ~625 kDa. It is composed of typically 17 different protein subunits (Figure 1) and organizes into a membrane-extrinsic F_1_ catalytic and membrane-embedded F_o_ domains, which are connected by a peripheral and central stalk (Allegretti et al., 2015; Morales-Rios et al., 2015a; Zhou et al., 2015). The matrix-oriented F_1_ is composed of a prominent (αβ)_3_ catalytic head into which the γ*δε* central stalk rotor penetrates. The F_o_ *c*-ring typically consists of identical *c* subunits (subunit *9* in yeast). Together with subunit *a*, the *c*-ring shuttles protons across the membrane. The F_o_ domain further consists of subunits *8* (alias *A6L*), *b (4* in yeast*), f*, *d, F6* (*h* in yeast), and *OSCP* that together form the peripheral stalk connecting the catalytic head with the membrane stator. Three mitochondria-specific subunits, *e, g, k*, induce either directly or indirectly the formation of ATP synthase dimers (Hahn et al., 2016) that self-assemble in longer ribbons important for cristae formation (Parsons, 1963; Strauss et al., 2008).

The ATP synthase harbors a unique rotary mechanism driven by the *pmf* to translocate ions through F_o_, to generate rotation of its rotor and transmit torque into the F_1_ catalytic head where finally ATP is synthesized and released. Subunit *a* provides a pathway that involves a number of hydrophilic amino acids, which allows protons to enter from the IMS (Figure 1). Approximately in the middle of the membrane the proton can bind to a highly conserved acidic residue of subunit *c* helix 2 (*c*H2) (*c*E59 in *H. sapiens*) located at the outer surface of the *c*-ring. It has been suggested that the binding of a proton on this carboxylate residue disrupts a previously established electrostatic interaction of *c*E59 with a highly conserved, positively charged arginine residue in subunit *a* membrane helix 5 (*a*H5) (*a*R159 in *H. sapiens*; Vik and Antonio, 1994; Junge et al., 1997; Pogoryelov et al., 2010; Guo et al., 2017). This arginine acts as an electrostatic separator between the proton pathway from the IMS to the middle of the membrane and a second, spatially separated pathway that allows incoming protons still bound on the *c*-ring glutamate to be released into the matrix (Mitome et al., 2010). The operation direction of this process is primarily driven by the ion gradient that causes a ratchet type mechanism of the neutralized *c*-ring glutamate in the hydrophobic membrane, which is energetically unfavorable and does not allow the back stepping without externally applied force (Vik and Antonio, 1994; Junge et al., 1997). After an almost complete revolution of the *c*-ring, the glutamate is deprotonated in the aqueous exit channel (Pogoryelov et al., 2010). This channel is formed by hydrophilic residues of the *c*-ring/subunit *a* interface at the matrix side through which the protons can reach the matrix (*n*-Side) (Allegretti et al., 2015; Morales-Rios et al., 2015a; Zhou et al., 2015; Hahn et al., 2016; Guo et al., 2017). The *c*-ring is tightly bound to the central stalk subunits γ*δε*, of which subunit γ protrudes into the F_1_ catalytic head, which induces cyclic conformational changes when rotating (Abrahams et al., 1994). Consequently, ADP and P_i_ are sequentially converted at the catalytic sites of the three subunits β, according to the binding change mechanism (Boyer, 1997). Cryo-EM structures of the bovine *Bos taurus* and yeasts *Yarrowia lipolytica* and *Saccharomyces cerevisiae* F_1_F_o_ ATP synthases, that are basically of the same subunit composition and structural construction as the human enzyme, have been described recently [15–17]. These structures show a very similar overall architecture and differ only with respect to the subunit *c* (*9*) stoichiometry (8 in mammals, 10 in yeasts), the loss of the dimerization domain subunits (*e*/*g*/*k*) during purification (yeast) and the non-essential and yeast specific subunits *i* and *k* (Zhou et al., 2015; Hahn et al., 2016; Guo et al., 2017). It therefore has become feasible to build reliable structural models of the membrane domain (F_o_) of the eukaryotic, mitochondrial, ATP synthase (Figure 1), and to map the human disease-causing mutations at the molecular level within the ATP synthase structure and to pin-point their potentially adverse effects on the above-described mechanism.

## Yeast and human cellular models of mtDNA diseases

Human cells contain up to thousands copies of mtDNA (Miller et al., 2003). Mutations in this DNA are highly recessive and usually co-exist with wild type mtDNA molecules, a situation referred to as heteroplasmy. A mutational load above 60% is usually required to induce a clinical phenotype (Stewart and Chinnery, 2015). Given the high mutational rate of the mitochondrial genome and the presence of numerous family or population-specific polymorphisms, it can be difficult to distinguish between a neutral mtDNA variant and a disease-causing mutation. Additionally, the effects of deleterious mtDNA mutations might be exacerbated by mtDNA nucleotide changes that are not pathogenic *per se* and by unknown factors in nuclear genetic background, i.e., the so-called modifier genes (Cai et al., 2008; Swalwell et al., 2008). These features make it difficult from patient's cells and tissues to precisely know how specific mtDNA mutations influence oxidative phosphorylation.

To better characterize the effects of mtDNA mutations, homoplasmic cell lines, i.e. with a 100% mutational load, in a defined nuclear genetic background are required. To this end King and Attardi (King and Attardi, 1989) developed an approach that used cybrid (cytoplasmic hybrid) cell lines obtained by fusing enucleated cytoplasts from patient's cells with cells lacking mtDNA (ρ^0^). This approach was used to evaluate the bioenergetics consequences of 11 ATP6 mutations (Trounce et al., 1994; Majander et al., 1997; Nijtmans et al., 2001; Carrozzo et al., 2004; Mattiazzi et al., 2004; Pallotti et al., 2004; Jonckheere et al., 2008; Sikorska et al., 2009; Aure et al., 2013; Blanco-Grau et al., 2013; Lopez-Gallardo et al., 2014; Hejzlarova et al., 2015; Wen et al., 2016). Another approach exploits unique features of *S. cerevisiae*. Mitochondria from this single-celled fungus and humans show many similarities (Steinmetz et al., 2002; Prokisch et al., 2004; Reinders et al., 2006; Pagliarini et al., 2008; Rhee et al., 2013), and mitochondrial genetic transformation can be achieved in this yeast in a highly controlled fashion, by the biolistic delivery into mitochondria of *in-vitro*-made mutated mtDNA fragments, followed by their integration into wild type mtDNA by homologous DNA recombination (Bonnefoy and Fox, 2001). Being unable to stably maintain heteroplasmy (Okamoto et al., 1998), it is easy to obtain yeast homoplasmic populations where all mtDNA molecules carry a mutation of interest. Owing to its good fermenting capacity, yeast models of human mitochondrial diseases can be kept alive when provided with sugars like glucose even when oxidative phosphorylation is completely inactivated (Baile and Claypool, 2013; Lasserre et al., 2015). This approach was used to investigate the impact on ATP synthase of nine ATP6 mutations identified in patients (Rak et al., 2007; Kucharczyk et al., 2009a,b,c, 2010, 2013; Kabala et al., 2014; Lasserre et al., 2015; Niedzwiecka et al., 2016; Wen et al., 2016).

## Pathogenic mutations in ATP8 and ATP6

**Figure 2:**
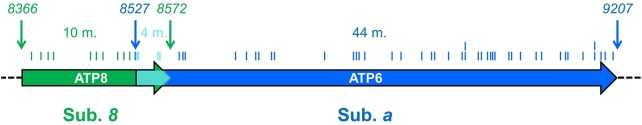
ATP6 and ATP8 genes and sites of pathogenic mutations. The two coding sequences overlap, between positions 8527 and 8572 (cyan) of the human mitochondrial genome. Start and end nucleotide numbers are indicated. The number of mutations (m) identified in patients and their positions are indicated. These mutations are listed in Table 2.

**Figure 3:**
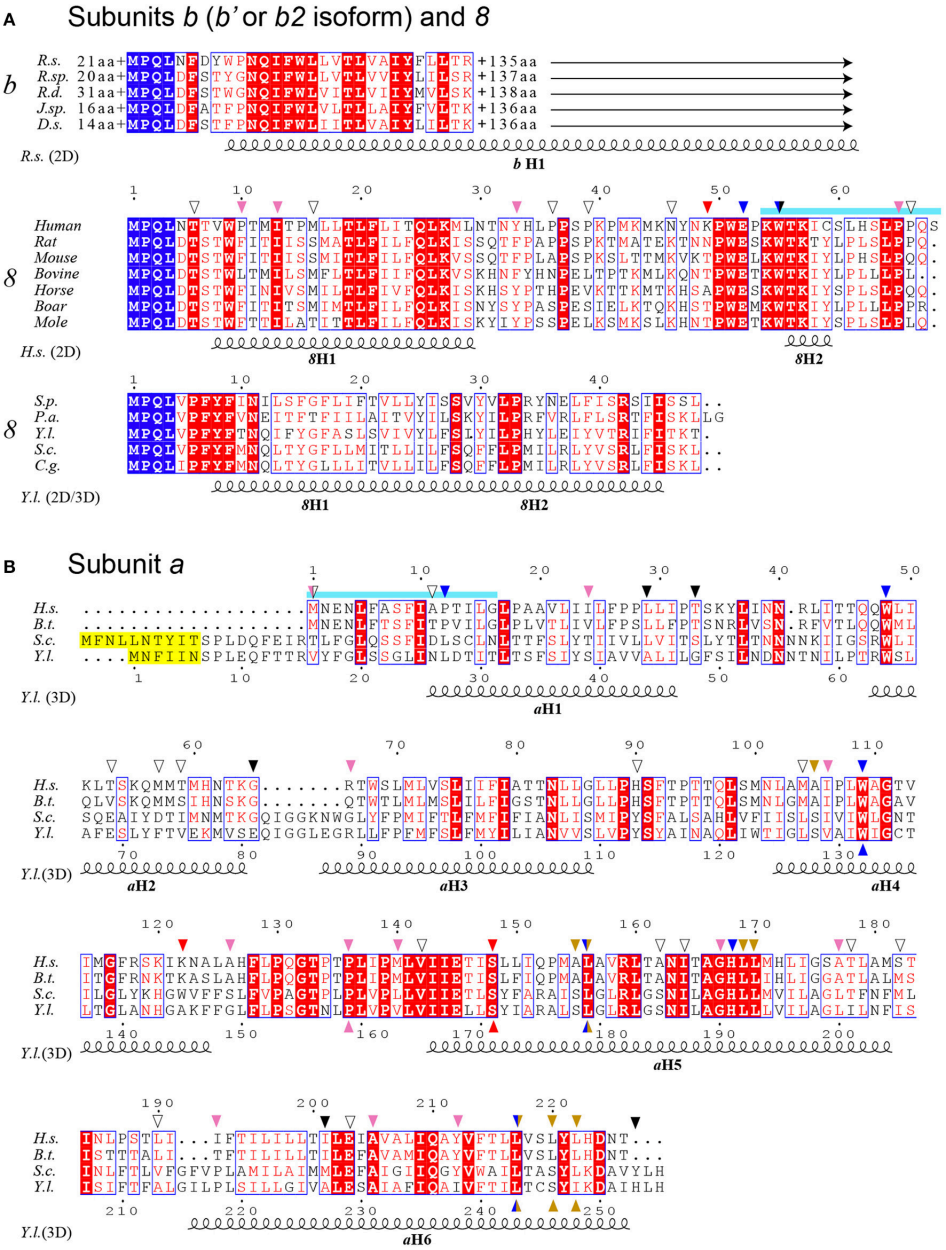
Sequence alignment of subunits *a, b* and *8* from a selected range of species. **(A)** Alignment of bacterial subunits *b* (*b*′ or *b2* isoform) and mitochondrial subunits *8*. The sequences of subunit *b* are from *Rhodobacter sphaeroides* (*R.s*.)*, Ruegeria* sp. (*R.sp*)*, Roseobacter denitrificans* (*R.d*.), *Jannaschia* sp. (*J.sp*) *and Dinoroseobacter shibae* (*D.s*.). The length of N- and C-termini extensions are given. The sequences of subunit *8* are from a selection of mammals and the yeast species *Schizosaccharomyces pombe (S.p.), Podospora anserina (P.a.), Yarrowia lipolytica* (*Y.l*.)*, Saccharomyces cerevisiae* (*S.c*.) and *Candida glabrata* (*C.g*.). **(B)** Alignment of subunit *a*. *Homo sapiens (H.s.), Bos taurus* (*B.t*.). The sequences corresponding to overlapping ATP8 and ATP6 genes are marked in cyan as in Figure 2. Peptide sequences removed in the mature form of yeast subunit *a* are marked in yellow. At the top and bottom, the arrows mark the locations of the human mutations (Table 2) and the mutations modeled in *S*.c., respectively. The arrows are colored according to Figure 4. At the bottom, the secondary structural elements are drawn according to PSIPRED prediction (2D) and a cryo-EM structure (3D) (Hahn et al., 2016).

Subunits *8* and *a* are synthesized from a bi-cistronic mRNA unit (Figure 2). The two genes show a 46 nucleotide overlap. Thus, mutations in this unit can affect either subunit *a* or *8*, or both. Currently, 36 different ATP8 and ATP6 mutations with a confirmed or suspected pathogenic character are recorded in MITOMAP database (Figure 3). We here review 22 additional mutations found in literature. The nucleotide and amino acid changes induced by these mutations, and what is known about their functional and clinical consequences is summarized in Table 2.

**Table 2:** Pathogenic mutations in ATP8 and ATP6 genes, associated disease(s)/syndrome(s), number of cases (N), patient's age (year), heteroplasmy (H), pathogenicity (PG), and ATP synthase activities and mitochondrial morphology.

**mtDNA mutation, m**.	**Protein mutation**	**Disease(s) syndrome(s)**	**N**	**Age (y)**	**H %**	**ATP synthase**		**ROS/Mitochondria morphology**	**References**	**PG**
						**Activity**	**Assembly/Stability**			
8381A>G	*8*T6A	MIDD, LVHT	2	38,57	99	nd	nd	nd/Abnormal	Perucca-Lostanlen et al., 2000; Finsterer et al., 2004	U
8393C>T	*8*P10S	Epilepsy with brain pseudoatrophy	1	10	<99	nd	nd	nd/nd	Galimberti et al., 2006	U
8403T>C	*8*I13T	Episodic weakness	1	8	99	Normal	Normal	Higher/nd	Aure et al., 2013	U
8411A>G	*8*M16V	Neurologic disorder with blindness	1^†^	10	97	nd	nd	nd/nd	Mkaouar-Rebai et al., 2010	U
8463A>G	*8*Y33C	Schizophrenia	1	nd	100	nd	nd	nd/nd	Sequeira et al., 2015	U
8472C>T	*8*P36L	Autism	3	4–8	100	nd	nd	nd/nd	Piryaei et al., 2012	U
8481C>T	*8*P39L	ToF	1	1	100	nd	nd	nd/nd	Tansel et al., 2014	U
8502A>T	*8*N46I	MTLE-HS	40	Adults	11–36	nd	nd	nd/nd	Gurses et al., 2014	S
8510A>G	*8*K49E	Schizophrenia	1	nd	100	nd	nd	nd/nd	Ueno et al., 2009	U
8519G>A	*8*E52K	Schizophrenia	1	nd	100	nd	nd	nd/nd	Sequeira et al., 2015	U
8528T>C	*8*W55R *a*M1T	Infantile cardiomyopathy	5, 3^†^	0.1–4	90–98	Reduced	nd	nd/nd	Ware et al., 2009; Imai et al., 2016	S
8529G>A	*8*W55X *a*M1I	Apical hypertrophic cardiomyopathy and neuropathy	1	16	90	Reduced	Defective	nd/nd	Jonckheere et al., 2008	S
8558C>T	*8*P65S *a*A11V	LVHC	1	0.2	nd	nd	nd	nd/nd	Tang et al., 2010	C
8561C>G	*8*P66A *a*P12R	Ataxia, neuropathy, diabetes mellitus	2	59, 64	99	Reduced	Defective	nd/nd	Kytovuori et al., 2016	S
8527A>G	*a*M1V	Neuromuscular Disorder	1	7	nd	nd	nd	nd/nd	Felhi et al., 2016	U
8597T>C	*a*I24T	MILS	1	2	95	nd	nd	nd/nd	Tsai et al., 2012	U
8611insC	*a*L29PfsX36	Ataxia, Encephalopathy	1	4	60–80	Reduced	Defective	nd/Abnormal	Jackson et al., 2017	S
8618insT	*a*T33HfsX32	NARP	1	40	85	nd	Defective	nd/nd	Lopez-Gallardo et al., 2009	U
8668T>C	*a*W48R	LHON	1	Adult	99	nd	nd	nd/nd	Kumar et al., 2010	U
8684C>T	*a*T53I	Autism associated Ovarian insufficiency LHON	1 7 1	4–8 25 Adult	100	nd	nd	nd/nd	Piryaei et al., 2012 Venkatesh et al., 2010 Kumar et al., 2010	U
8697G>A	*a*M57I	Autism associated LHON	5	4–8	100	nd	nd	nd/nd	Kumar et al., 2010; Piryaei et al., 2012	U
8701A>G	*a*T59A	Autism associated	1	4–8	100	nd	nd	nd/nd	Piryaei et al., 2012	U
8719G>A	*a*G65X	Suspected myopathy	1	nd	<99	nd	nd	nd/nd	Tang et al., 2013	U
8723G>A	*a*R66Q	Schizophrenia	1	nd	100	nd	nd	nd/nd	Ueno et al., 2009	U
8794C>T	*a*H90Y	Schizophrenia	1	nd	100	nd	nd	nd/nd	Sequeira et al., 2015	U
8836A>G	*a*M104V	LHON Autism associated	1 3	11 4–8	100	nd	nd	nd/nd	Abu-Amero and Bosley, 2006 Piryaei et al., 2012	U
8839G>C	*a*A105P	NARP	1	57	21–88	Normal	nd	nd/nd	Blanco-Grau et al., 2013	S
8843T>C	*a*I106T	Schizophrenia	2	nd	100	nd	nd	nd/nd	Ueno et al., 2009; Sequeira et al., 2015	U
8851T>C	*a*W109R	FBSN	2	3	87–99	Reduced	Defective	nd/Abnormal	De Meirleir et al., 1995; Honzik et al., 2013; Kucharczyk et al., 2013	S
8890A>G	*a*K122E	MS	1	18	35–38	nd	nd	nd/nd	Ye et al., 2013	U
8902G>A	*a*A126T	Schizophrenia	1	nd	100	nd	nd	nd/nd	Ueno et al., 2009	U
8932C>T	*a*P136S	Neuromuscular Disorder	1	7	100	Reduced	Defective	nd/nd	Petros et al., 2005; Felhi et al., 2016; Niedzwiecka et al., 2016	S
8945T>C	*a*M140T	Schizophrenia	1	nd	100	nd	nd	nd/nd	Ueno et al., 2009	U
8950G>A	*a*V142I	LHON plus dystonia	1	23	nd	Reduced	nd	nd/nd	Abu-Amero and Bosley, 2005	U
8969G>A	*a*S148N	MLASA Nephropathy	1 1	6 14	60–90	Reduced	Defective	Higher/nd	Burrage et al., 2014 Wen et al., 2016	C
8989G>C	*a*A155P	NARP	1	53	33–94	Reduced	nd	nd/nd	Duno et al., 2013; Wen et al., 2016	S
8993T>G	*a*L156R	NARP/MILS	53, 10^†^	0.1–53	13–99	Reduced	Normal	Higher/Abnormal	Holt et al., 1990; Tatuch et al., 1992; Ciafaloni et al., 1993; Puddu et al., 1993; Trounce et al., 1994; Houstek et al., 1995; Uziel et al., 1997; Vazquez-Memije et al., 1998; Baracca et al., 2000, 2007; Garcia et al., 2000; Nijtmans et al., 2001; Carrozzo et al., 2004; Mattiazzi et al., 2004; Pallotti et al., 2004; Sakai et al., 2004; Enns et al., 2006; Morava et al., 2006; Rojo et al., 2006; Sgarbi et al., 2006; Cortes-Hernandez et al., 2007; Rak et al., 2007	C
8993T>C	*a*L156P	NARP/MILS	35, 4^†^	0.1–77	20–96	Reduced	Defective	Higher/Normal	De Vries et al., 1993; Santorelli et al., 1994; Fujii et al., 1998; Vazquez-Memije et al., 1998; Vilarinho et al., 2001; Hurvitz et al., 2002; Pallotti et al., 2004; Morava et al., 2006; Baracca et al., 2007; Craig et al., 2007; Debray et al., 2007; Kucharczyk et al., 2009a; Kara et al., 2012; Aure et al., 2013; Martikainen et al., 2015	C
9011C>T	*a*A162V	LHON	1	34	100	nd	nd	nd/nd	Shidara and Wakakura, 2012	U
9016A>G	*a*I164V	LHON	1	Adult	100	nd	nd	nd/nd	Povalko et al., 2005	U
9025G>A	*a*G167S	MILS-like, NARP	2, 1^†^	0.2, nd	100	nd	nd	nd/nd	Lopez-Gallardo et al., 2014	U
9029A>G	*a*H168R	LHON-like	1	38	95–99	Reduced	nd	Higher/nd	Lopez-Gallardo et al., 2014	S
9032T>C	*a*L169P	NARP	1	16	70–90	Reduced	nd	Higher/nd	Lopez-Gallardo et al., 2014	S
9035T>C	*a*L170P	SCA	21	4–48	90–99	Reduced	nd	Higher/nd	Sikorska et al., 2009; Pfeffer et al., 2012	C
9055G>A	*a*A177T	Schizophrenia	3	nd	100	nd	nd	nd/nd	Sequeira et al., 2015	U
9058A>G	*a*T178A	LVHT	1	nd	nd	nd	nd	nd/nd	Tang et al., 2010	U
9071C>T	*a*S182L	Schizophrenia	1	nd	100	nd	nd	nd/nd	Ueno et al., 2009	U
9094C>T	*a*L190F	Ovarian insufficiency	5	25	nd	nd	nd	nd/nd	Venkatesh et al., 2010	U
9101T>C	*a*I192T	LHON	1	21	100	Reduced	nd	nd/nd	Lamminen et al., 1995; Majander et al., 1997	S
9127delAT	*a*I201PfsX2	NARP	1	18	10–82	Reduced	nd	nd/nd	Mordel et al., 2017	S
9134A>G	*a*E203G	MS with cardiomyopathy	1	nd	nd	Reduced	nd	nd/nd	Honzik et al., 2012	U
9139G>A	*a*A205T	LHON	2	30,45	nd	nd	nd	nd/nd	La Morgia et al., 2008	U
9160T>C	*a*Y212H	Schizophrenia	1	nd	100	nd	nd	nd/nd	Sequeira et al., 2015	U
9176T>G	*a*L217R	MILS	6, 3^†^	3–42	95–99	Reduced	Defective	Higher/Abnormal	Thyagarajan et al., 1995; Dionisi-Vici et al., 1998; Makino et al., 1998; Okamoto et al., 1998; Jacobs et al., 2005; Kucharczyk et al., 2009b; Ronchi et al., 2011; Verny et al., 2011; Synofzik et al., 2012; Aure et al., 2013	C
9176T>C	*a*L217P	MILS, Periodic paralyzes, CMT, HSP	19, 4^†^	1–59	90–99	Reduced	Defective	Higher/Abnormal	Thyagarajan et al., 1995; Dionisi-Vici et al., 1998; Makino et al., 1998; Okamoto et al., 1998; Jacobs et al., 2005; Kucharczyk et al., 2010; Ronchi et al., 2011; Verny et al., 2011; Synofzik et al., 2012; Aure et al., 2013	C
9185T>C	*a*L220P	Periodic paralyzes, Ataxia, MILS, CMT, MNS, SCA	61, 4^†^	2–58	73–99	Reduced	Defective	nd/Abnormal	Moslemi et al., 2005; Castagna et al., 2007; Childs et al., 2007; Saneto and Singh, 2010; Pfeffer et al., 2012; Pitceathly et al., 2012; Aure et al., 2013; Brum et al., 2014; Kabala et al., 2014	C
9191T>C	*a*L222P	MILS	1^†^	2	90–94	Reduced	nd	nd/Abnormal	Moslemi et al., 2005; Kabala et al., 2014	S
9205delTA	*a*STOP elimination	Encephalopathy/Lactic acidosis	3, 1^†^	Adult, nd	98–99	Reduced	nd	nd/Abnormal	Seneca et al., 1996; Jesina et al., 2004; Hejzlarova et al., 2015	C

† *–nr of died patients, nd, no data; PG, pathogenicity; C, confirmed; S, suspected; U, unknown; mutations not present in the MITOMAP list are underlined*.

### Mutations affecting only subunit *8*

Ten mutations affecting only subunit *8* were identified in patients presenting with various disorders: epilepsy [**m.8502A**>**T** (*8*N46I)] (Gurses et al., 2014); LVHT or MIDD **[m.8381A**>**G** (*8*T6A)] (Perucca-Lostanlen et al., 2000; Finsterer et al., 2004); brain pseudo-atrophy, episodic weakness and neurological disorders [**m.8393C**>**T** (*8*P10S) (Galimberti et al., 2006), **m.8403T**>**C** (*8*I13T) (Aure et al., 2013), and **m.8411A**>**G** (*8*M16V) (Mkaouar-Rebai et al., 2010)], heart problems [**m.8481C**>**T** (*8*P39L)] (Tansel et al., 2014); schizophrenia [**m.8463A**>**G** (*8*Y33C)**, m.8510A**>**G** (*8*K49E) and **m.8519G**>**A** (*8*E52K)] (Ueno et al., 2009; Sequeira et al., 2015); and autism [**m.8472C**>**T** (*8*P36L)] (Piryaei et al., 2012).

### Mutations affecting both subunits *8* and *a*

Three mutations affecting both subunits *8* and *a* [**m.8528T**>**C** (*8*W55R + *a*M1T), **m.8529G**>**A** (*8*W55STOP + *a*M1I), **m.8558C**>**T** (*8*P65S + *a*A11V)] were identified in patients suffering from severe cardiomyopathies (Jonckheere et al., 2008; Ware et al., 2009; Tang et al., 2010; Imai et al., 2016). A fourth mutation affecting both proteins [**m.8561C**>**G** (*8*P66A + *a*P12R)] was detected in individuals with features of cerebellar ataxia, peripheral neuropathy and diabetes mellitus (Kytovuori et al., 2016).

### Mutations affecting only subunit *a*

#### Most frequent mutations

- Two mutations at the same amino acid position of subunit *a*, **m.8993T**>**G** (*a*L156R) and **m.8993T**>**C** (*a*L156P), were identified in numerous patients presenting with the NARP or MILS syndrome depending on the mutation load (Uziel et al., 1997; Jonckheere et al., 2012). The first one was consistently found to severely compromise mitochondrial ATP production, with deficits of up to 90%. While most studies concluded this was due to a block in proton translocation, some suggested a less efficient coupling or defects in the assembly/stability of ATP synthase (Tatuch et al., 1992; Trounce et al., 1994; Houstek et al., 1995; Vazquez-Memije et al., 1998; Garcia et al., 2000; Nijtmans et al., 2001; Carrozzo et al., 2004; Mattiazzi et al., 2004; Pallotti et al., 2004; Morava et al., 2006; Sgarbi et al., 2006; Baracca et al., 2007; Cortes-Hernandez et al., 2007). Although its pathogenic character is firmly established, the second mutation has less severe consequences on ATP synthase with a 70% drop in ATP production mainly because of a less efficient assembly or diminished stability of subunit *a* (Vilarinho et al., 2001; Morava et al., 2006; Craig et al., 2007; Kucharczyk et al., 2009a; Aure et al., 2013). In addition to bioenergetic deficits, the two mutations lead to an enhanced production of ROS and aberrant mitochondrial morphologies that may contribute to the disease process as well.
- Similar diseases were associated with two mutations at amino acid position 217, **m.9176T**>**G** (*a*L217R), and **m.9176T**>**C** (*a*L217P). The first one is extremely detrimental with a block in subunit *a* assembly that leads to extreme clinical phenotypes when highly abundant in cells and tissues. The second one does not obviously compromise assembly of subunit *a* indicating that it affects the functioning of ATP synthase.
- The **m.9185T**>**C** (*a*L220P) mutation was identified in individuals presenting with MILS, MNS, periodic paralyzes, spinocerebellar ataxia syndromes (SCA) or CMT (Castagna et al., 2007; Childs et al., 2007). Biochemical analyses revealed a substantial drop (50–90%) in ATP production, and study in yeast indicated that this mutation compromises the functioning of ATP synthase (Kabala et al., 2014).

#### Other, less frequent, mutations in subunit *a*

- Eight additional mutations in subunit *a* [**m.8597T**>**C** (*a*I24T), **m.8618insT**, **m.8839G**>**C** (*a*A105P), **m.8989G**>**C** (*a*A155P), **m.9025G**>**A** (*a*G167S), **m.9032T**>**C** (*a*L169P), **m.9127delAT** (*a*I201PfsX2) and **m.9191T**>**C** (*a*L222P)] were identified in patients presenting with NARP or MILS disease. Although they were thus far found only in a limited number of individuals, biochemical investigations indicated that they have detrimental consequences for ATP synthase. For instance, the **m.9191T**>**C** was shown to dramatically affect incorporation of subunit *a* in the yeast enzyme (Kabala et al., 2014). These mutations are thus most likely pathogenic.
- The **m.8969G**>**A** (*a*S148N) mutation was found in a patient presenting with MLASA (Burrage et al., 2014) and a 14-year-old Chinese girl diagnosed with a severe nephropathy (Wen et al., 2016). Biochemical investigations in yeast and human cells revealed a block in the transfer of protons through the F_o_.
- The **m.8851T**>**C** (*a*W109R) and **m.8890A**>**G** (*a*K122E) mutations were identified in patients presenting with FBSN and MS (De Meirleir et al., 1995; Honzik et al., 2013; Ye et al., 2013). Studies with yeast revealed that **m.8851T**>**C** leads to major drops (95%) in mitochondrial ATP synthesis owing to a block in F_o_-mediated proton transfer (Kucharczyk et al., 2013). The **m.9134A**>**G** (*a*E203G) was identified in patient suffering from MS accompanied with cardiomyopathy (Honzik et al., 2012).
- The **m.9035T**>**C** (*a*L170P) was identified at high (>90%) mutation load in 21 ataxia patients (Sikorska et al., 2009; Pfeffer et al., 2012). Cybrids carrying this mutation had reduced ATP levels (40–50% vs. controls) and produced 5–7 times more ROS than control cells. Another mutation **m.8611insC** (*a*L29PfsX36) was found in patient presenting ataxia with encephalomyopathy (Jackson et al., 2017).
- Ten mutations [**m.8668T**>**C** (*a*W48R), **m.8684C**>**T** (*a*T53I), **m.8697G**>**A** (*a*M57I), **m.8836A**>**G** (*a*M104V), **m.8950G**>**A** (*a*V142I), **m.9011C**>**T** (*a*A162V), **m.9016A**>**G** (*a*I164V), **m.9029A**>**G** (*a*H168R), **m.9101T**>**C** (*a*I192T), and **m.9139G**>**A** (*a*A205T)] were identified in patients presenting with LHON, a disease caused by defects in the retinal ganglion cells and optic nerve that lead to blindness. Two of them (**m.9029A**>**G** and **m.9101T**>**C**) were shown to compromise oxidative phosphorylation by a yet-unknown mechanism (Lamminen et al., 1995; Lopez-Gallardo et al., 2014).
- The **m.8932C**>**T** (*a*P136S) and **m.8527A**>**G** (*a*M1V) were identified in children with neuromuscular disorders (Felhi et al., 2016). The first one was also identified in prostatic cancer cells (Petros et al., 2005). In a yeast model of this mutation, ATP synthase assembly/stability was found substantially affected (Niedzwiecka et al., 2016).
- The **m.9205delTA** (*a*STOP elimination) mutation was identified in patients with a severe encephalopathy leading to premature death. Since the stop codon of ATP6 overlaps with the start codon of COX3, the expression of both genes is compromised, which results in a lower content in complex IV and ATP synthase (Jesina et al., 2004; Hejzlarova et al., 2015).
- The **m.8719G**>**A** (*a*G65STOP) and **m.9058A**>**G** (*a*T178A) mutations were identified in patients presenting with LVHT (Tang et al., 2010, 2013). Their biochemical consequences are still unknown.
- Ten mutations [**m.8701A**>**G** (*a*T59A), **m.8723G**>**A** (*a*R66Q), **m.8794C**>**T** (*a*H90Y), **m.8843T**>**C** (*a*I106T), **m.8902G**>**A** (*a*A126T), **m.8945T**>**C** (*a*M140T), **m.9055G**>**A** (*a*I177T), **m.9071C**>**T** (*a*S182L), **m.9094C**>**T** (*a*L190F), and **m.9160T**>**C** (*a*Y212H)] were found in patients with autism or schizophrenia. Their consequences on ATP synthase has not yet been investigated.

It is puzzling that mutations that cluster in specific regions of ATP6 or ATP8 give rise to such a wide variety of clinical symptoms (Table 1). These phenotypic differences may be due to other unknown genetic variations in patients within mitochondrial or nuclear DNA that could exacerbate or attenuate the consequences on health of defects in ATP synthase subunits. Another important source of variability in the clinical outcome likely resides in the levels of heteroplasmy and different distributions of mtDNA mutations in cells and tissues. Furthermore, in addition to a lack of ATP, defects in ATP synthase may have multiple secondary effects, such as increased production of ROS and changes in upstream metabolic processes (Korshunov et al., 1997) that together will influence the disease process unpredictably.

## Topology within the F_o_ of mutations in subunits *a* and *8*

**Figure 4:**
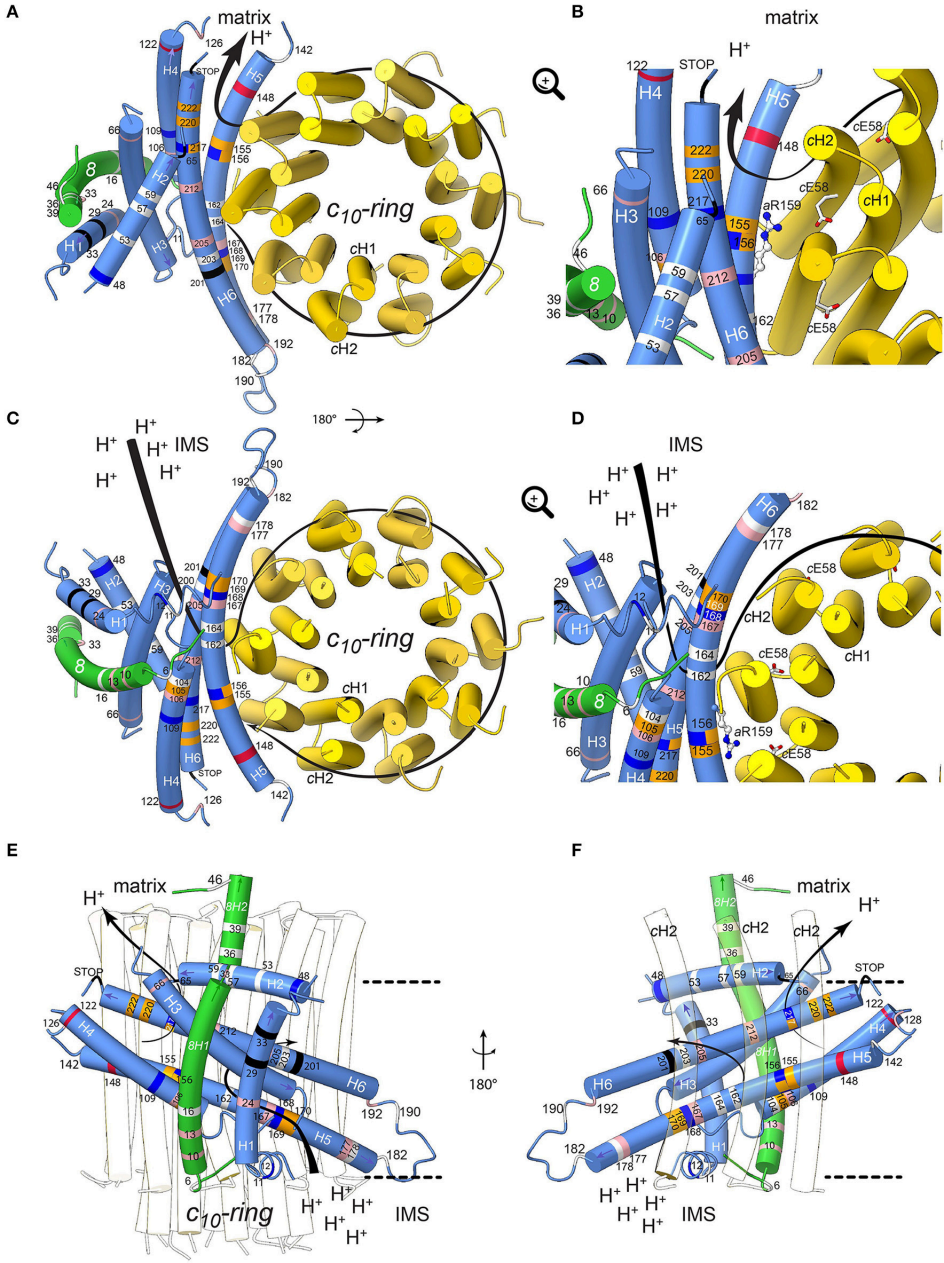
Positions of human neurodegenerative disease-causing mutations in the structure of the yeast mitochondrial ATP synthase subunits *a* and *8* (human *A6L*). The views are from the matrix **(A,B)** and from the IMS **(C,D)** with detailed views in the IMS entry channel **(B)** and in the matrix exit channel **(D)**. On **(E,F)**, showing views along the membrane plane from outside the F_o_ stator and from the *c*-ring, respectively, the membrane borders are indicated as black lines. Subunits *a, 8*, and *c* are shown in blue, green, and yellow, respectively. The model is based on structural data from the *Y. lipolytica* and *S. cerevisiae* structures (Hahn et al., 2016; Guo et al., 2017). The positions of mutations, which have been found in human neurodegenerative diseases, are marked in white, red, blue, pink, orange and black for hydrophobic, negatively and positively charged, uncharged polar, proline and special residues, respectively. The mutations are labeled according to their positions in human subunit *a* listed in Table 2. The arrows indicate the path of protons in ATP synthesis direction. The conserved arginine (*h.s. a*R159, *S.c. a*R186) on helix *a*H5 is indicated by stick model; its orientation is randomly chosen. The figure was drawn with UCSF ChimeraX (Goddard et al., 2017).

To define the topology of the ATP6 and ATP8 mutations identified in patients, we used the recently published structures of *Y. lipolytica* and *S. cerevisiae* ATP synthases (Hahn et al., 2016; Guo et al., 2017); the complete model of subunits *a, 8*, and the *c*-ring is shown in Figure 4. The amino acid alignments in Figure 3 establish the correspondences with human subunits *a* and *8* amino acids.

### Subunit *a* mutations

Helix 5 of subunit *a* (*a*H5) is kinked due to the presence of proline at position 153, a residue well-known for its propensity to bend or break alpha helices owing to its inability to participate fully in protein backbone hydrogen bonding. *a*P153 enables *a*H5 to follow the curvature of the *c*-ring and seal the two hydrophilic pockets that connect the *a*/c-ring interface to the intermembrane and matrix spaces. Five substitutions from hydrophobic alanine or leucine residues into proline are located on *a*H5 (*a*H5) in proximity to the essential *a*R159 residue (*a*A105P, *a*A155P and *a*L156P) that faces the proton binding glutamate of the *c*-ring, or close to *a*H168/*a*E203 in the proton entry channel (*a*L169P, *a*L170P). These mutations may distort or break *a*H5. Those at positions 155 and 156 at one helix turn from *a*R159 may compromise the ion translocation mechanism, for example by ion short circuiting (Mitome et al., 2010) or by preventing *a*R159 to interact with the *c*-ring glutamate due to its structurally shifted position. On *a*H6, the *a*L217P, *a*L220P, and *a*L222P mutations are on the edge of the exit channel close to the matrix. Their severe functional consequences possibly result from a change in the topology of the nearby *a*D224 residue that was suggested to be of critical importance for the exit of protons toward the mitochondrial matrix (Guo et al., 2017). Similarly, the *a*P136S change in the loop connecting *a*H4 and *a*H5 possibly alters the accessibility of protons in this region of subunit *a*.

The extremely detrimental consequences of *a*L217R on ATP synthase assembly/stability (Kucharczyk et al., 2009b) possibly results from the inability of subunit *a* to pack tightly owing to replacement of a hydrophobic residue with a bulkier and positively charged one within the membrane. The absence of proton conduction induced by the *a*L156R mutation at the *a*/*c*-ring interface (Rak et al., 2007), without any defect in ATP synthase assembly, may be caused by the inability of protons to exit from the ring or by electrostatic or steric hindrance that prevent rotation of the ring. Being located near the N-terminal side of *a*H5, the block in proton translocation induced by the *a*S148N mutation (Wen et al., 2016) possibly results from obstruction of the proton exit pore. The detrimental consequences of *a*H168R (Lopez-Gallardo et al., 2014) are not surprising considering its location in the *p*-side cleft in the proximity of the *c*-ring. This positive charge cuts off the connection of the *p*-side to the *c*-ring. A similar effect on the *n*-side of the membrane may result from the *a*W109R, where *a*H5 and *a*H6 diverge. A mutation at this position has an impact in proton translocation without impacting ATP synthase assembly/stability (Kucharczyk et al., 2013), indicating that this location of subunit *a* is close to the pathway along which protons are evacuated into the matrix. The clinical consequences of *a*K122E probably also result from a less efficient proton conduction toward the matrix.

While *a*M140T, *a*G167S, *a*A177T, *a*I192T, *a*A205T, and *a*Y212H decrease the hydrophobicity of the *a*/*c*-ring interface, it is less obvious from our structural model to predict the consequences of other mutations that replace hydrophobic to non-charged hydrophilic residues (*a*I24T, *a*I106T, *a*A126T) or vice-versa (*a*T53I, *a*T59A, *a*T178A, *a*S182L) and those that lead to small hydrophobicity changes (*a*M57I, *a*M104V, *a*V142I, *a*A162V, *a*I164V, *a*L190F). However, meaningfully, most are within helices *a*H4-6 that are important for the movement of protons through F_o_.

### Subunit *8* mutations

The yeast subunit *8* has an overall kinked helical structure with a N-terminal transmembrane helix (*8*H1) and a short helix (*8*H2) exposed to the matrix. *8*H1 is flanked by subunit *i* and *a*H1 (Hahn et al., 2016; Guo et al., 2017). At the distal stator side, *8*H1 is exposed toward the dimer interface in a bent lipid bilayer region. At the base of the peripheral stalk, the C-terminus of *8*H2 is nestled inside the helical domain composed by subunits *b, d* and *f*. In vertebrates, no secondary structures are predicted in the C-terminal half of subunit *8* and subunit *f* displays only 18% identity with its yeast homolog, which explains why they could not be modeled from the bovine ATP synthase cryo-EM density map (Zhou et al., 2015). Subunit *8* shows at its N-terminus a conserved 4 amino acid motif (MPQL, Figure 3). This motif and *8*H1 share significant homologies with one of the two subunits *b* (isoform *b*′ or *b2*) from α-proteobacteria (Figure 3A), indicating that subunit *8* (*A6L*) is an evolutionary vestige of bacterial subunit *b* that remained mtDNA encoded (Hahn et al., 2016). Subunit *8* stabilizes in the membrane the helical N-terminal half of subunit *a* (Hahn et al., 2016). Four of the mutations identified in patients (*8*T6A, *8*P10S, *8*I13T, *8*M16V) cluster at the N-terminal region of helix *8*H1 in proximity to *a*H4, suggesting that they may affect the stability of subunits *a* and *8* (Hahn et al., 2016). The six other mutations identified in patients (*8*Y33C, *8*P36L, *8*P39L, *8*N46I, *8*K49E, *8*E52K) are in the matrix-exposed helix *8*H2. These mutations might affect the flexibility of the outer stalk, and thereby compromise the stability of F_o_ and/or, indirectly, the ion translocation mechanism or ATP synthase assembly process. This hypothesis is supported by the reduced functionality and stability of ATP synthase in these patients and by various studies on subunit *b* in the bacterial enzyme (Schneider and Altendorf, 1984, 1985; Wehrle et al., 2002; Greie et al., 2004).

## ATP synthase dimers and mitochondrial morphology

The mitochondrial ATP synthase exists as dimers (Schagger and Pfeiffer, 2000; Paumard et al., 2002) that associate into rows that contribute to cristae formation (Davies et al., 2011; Hahn et al., 2016). The mutations in subunits *8* and *a* often correlate with pathological forms of mitochondria cristae as for example seen in the Leigh syndrome (Kucharczyk et al., 2009b). The defects caused by these mutations therefore not only affect the ATP synthase function but they can also affect the assembly process. The reduced amount, or lack thereof, of native and completely assembled ATP synthase dimers would certainly affect cristae formation, which is crucial for the accommodation of the OXPHOS respiratory chain complexes and the ATP synthase. This explains some of the pathologic forms of mitochondria found in the diseases and syndromes described in this review.

## Conclusions

Diseases associated to mutations in the mitochondrial ATP6 and ATP8 genes are particularly challenging to study due to factors like heteroplasmy, complex inheritance, variable penetrance, and interactions with (e.g., nuclear) modifier genes, which makes it difficult to verify their pathogenicity. The possibility to create and keep alive homoplasmic strains of *S. cerevisiae* with defined mtDNA mutations in a controlled nuclear genetic background makes it possible to study their functional consequences. With the recently obtained cryo-EM structures of F_1_F_o_ ATP synthase from various mitochondrial origins it has become feasible to map mutations in ATP6 and ATP8 at the molecular level within the F_o_. These “open eyes” provide the chance for a completely new level of understanding of how the mutations may affect ATP synthase structure, assembly, and mechanism. This knowledge also enables to evaluate the observed pathogenic forms of mitochondrial morphology that are associated with these syndromes on the structural level, from the mutation at the molecular level to its associated consequences at the macroscopic scale of the organelle. The recent technical advances enabling the structural analysis of macromolecular complexes by cryo-EM (Kühlbrandt, 2014), the advent of complete structures of ATP synthases (Allegretti et al., 2015; Morales-Rios et al., 2015b; Zhou et al., 2015; Hahn et al., 2016; Sobti et al., 2016; Guo et al., 2017) and the availability of genetically approachable systems like *S. cerevisiae* are just the first steps in these new shoes; they will considerably improve the comprehension of human diseases associated to defects in this key mitochondrial enzyme. We are still at the beginning of understanding these complex processes.

## Author contributions

AD, AH, TM, DT-T, J-PdR, and RK discussed findings, analyzed literature and wrote the manuscript; AD, AH, and TM designed and made the Figures.

### Conflict of interest statement

The authors declare that the research was conducted in the absence of any commercial or financial relationships that could be construed as a potential conflict of interest.
